# Canagliflozin and fracture risk in individuals with type 2 diabetes: results from the CANVAS Program

**DOI:** 10.1007/s00125-019-4955-5

**Published:** 2019-08-10

**Authors:** Zien Zhou, Meg Jardine, Vlado Perkovic, David R. Matthews, Kenneth W. Mahaffey, Dick de Zeeuw, Greg Fulcher, Mehul Desai, Richard Oh, Roger Simpson, Nelson B. Watts, Bruce Neal

**Affiliations:** 1 The George Institute for Global Health, UNSW Sydney, Level 5, 1 King St, Newtown, Sydney, NSW 2042 Australia; 2 Department of Radiology, Ren Ji Hospital, School of Medicine, Shanghai Jiao Tong University, Shanghai, China; 3 Concord Repatriation General Hospital, Sydney, NSW Australia; 4 Oxford Centre for Diabetes, Endocrinology and Metabolism, University of Oxford, Oxford, UK; 5 Harris Manchester College, University of Oxford, Oxford, UK; 6 Stanford Center for Clinical Research, Department of Medicine, Stanford University School of Medicine, Stanford, CA USA; 7 University of Groningen, University Medical Center Groningen, Groningen, the Netherlands; 8 The Royal North Shore Hospital and University of Sydney, Sydney, NSW Australia; 9 Janssen Research & Development, LLC, Raritan, NJ USA; 10 University of Cincinnati, Cincinnati, OH USA; 11 Imperial College London, London, UK

**Keywords:** Canagliflozin, CANVAS Program, Fracture, Sodium glucose co-transporter 2 inhibitors, Type 2 diabetes mellitus

## Abstract

**Aims/hypothesis:**

An increased risk of fracture with canagliflozin vs placebo was reported from the CANagliflozin cardioVascular Assessment Study (CANVAS) Program, with heterogeneity of findings identified between the two trials that comprise the CANVAS Program, CANVAS and CANVAS-R. The objective of these analyses was to identify reasons for the possibly different effects on fracture observed between CANVAS and CANVAS-R.

**Methods:**

This study was an analysis of two highly similar trials, CANVAS and CANVAS-R, conducted in 10,142 individuals with type 2 diabetes and history or high risk of cardiovascular disease who received canagliflozin (pooled 100/300 mg once daily) or placebo. Outcomes assessed in this analysis were effects on adjudicated fractures overall and by type, location, association with a fall, dose and follow-up time.

**Results:**

A total of 496 participants recorded ≥1 fracture event during follow-up (15.40 vs 11.93 per 1000 patient-years with canagliflozin vs placebo; HR 1.26 [95% CI 1.04, 1.52]). There was significant heterogeneity in the effects on fracture (*p* = 0.005) between CANVAS (*n* = 4330: HR 1.55 [95% CI 1.21, 1.97]) and CANVAS-R (*n* = 5812: HR 0.86 [95% CI 0.62, 1.19]). The between-study heterogeneity in fracture risk was not clearly explained by differences in baseline characteristics, interactions of randomised treatment with participant characteristics, dose effects, duration of follow-up, metabolic effects, adverse events related to falls or adverse events possibly causing falls.

**Conclusions/interpretation:**

There was no evidence to explain clearly the fracture risk observed in the CANVAS Program or the heterogeneity in fracture risk between the two studies. The recently reported null result for fracture in the Canagliflozin and Renal Events in Diabetes with Established Nephropathy Clinical Evaluation (CREDENCE) trial suggests that the observed association in CANVAS is likely to be a chance finding, although an unidentified fall-related mechanism remains a possibility.

**Trial registration:**

ClinicalTrials.gov NCT01032629, NCT01989754.

**Electronic supplementary material:**

The online version of this article (10.1007/s00125-019-4955-5) contains peer-reviewed but unedited supplementary material, which is available to authorised users.

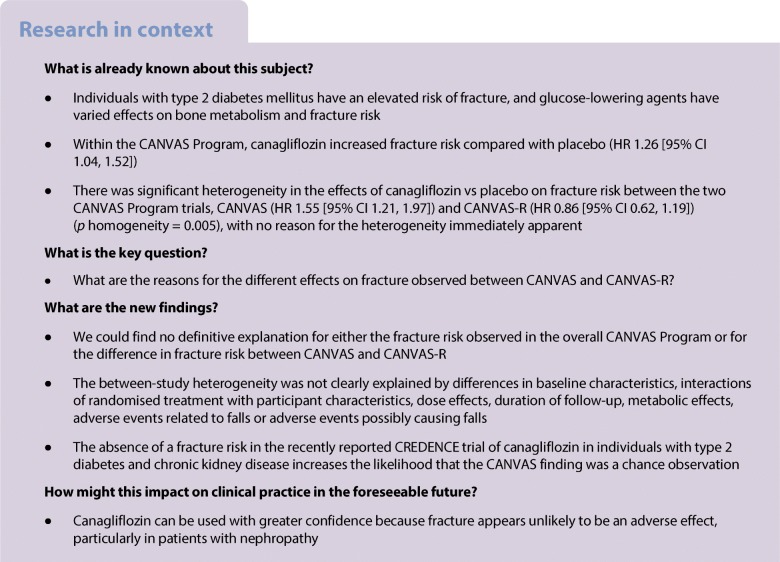



## Introduction

Individuals with type 2 diabetes mellitus have an elevated risk of fracture [1], though bone mineral density is typically preserved and may be increased in those with diabetes compared with those without diabetes [2, 3]. Glucose-lowering agents have varied effects on bone metabolism and fracture risk—thiazolidinediones lead to bone loss and increase the risk of fracture [4, 5], while sulfonylureas do not appear to influence bone health. Incretin-based medications (glucagon-like peptide-1 [GLP-1] receptor agonists and dipeptidyl peptidase-4 [DPP-4] inhibitors) and metformin may have protective effects on bone quality [6, 7].

Canagliflozin, a sodium glucose co-transporter 2 (SGLT2) inhibitor, acts to lower serum glucose by inhibiting renal tubular reabsorption of glucose. The CANagliflozin cardioVascular Assessment Study (CANVAS) Program [8] integrated data from two directly comparable, double-blind, randomised controlled trials (CANVAS and CANVAS-Renal [CANVAS-R]) [9, 10] carried out amongst people with type 2 diabetes mellitus at increased risk of cardiovascular disease. The trials were designed to complete simultaneously when a prespecified minimum number of cardiovascular events and a minimum follow-up time were achieved [11]. The prespecified strategy for analysis was to assess treatment effects in the combined data across the two studies to maximise statistical power, with assessment of the constancy of effects on the outcomes of interest across the trials by including an interaction term.

A routine interim review of the CANVAS trial performed in 2013 by the Data and Safety Monitoring Committee (DSMC) identified a higher incidence of fracture in the canagliflozin group compared with the placebo group (HR 1.51 [95% CI 1.04, 2.19]) [12]. Effects of canagliflozin on markers indicating some increase in bone turnover had been reported previously, as had some reduction of bone mineral density at the total hip, though not at other sites [13]. Following the DSMC report and further review, the US Food and Drug Administration (FDA) issued a labelled warning about elevated fracture risk in patients treated with canagliflozin. The other SGLT2 inhibitor that carries a warning related to fracture risk is dapagliflozin, which is restricted to the USA and applies only to individuals with moderate renal impairment [14].

CANVAS-R [10] was a second trial of canagliflozin performed in parallel to CANVAS [9]. The overall CANVAS Program findings comprising the integrated data from the two trials and the results from the individual trials were reported in 2017 [8]. Within the CANVAS Program, the effect of canagliflozin compared with placebo on fracture risk was attenuated (HR 1.26 [95% CI 1.04, 1.52]) compared with the initial report from CANVAS (HR 1.51 [95% CI 1.04, 2.19]) [8, 12]. Furthermore, there was significant heterogeneity in the effects of canagliflozin compared with placebo on fracture risk observed between CANVAS (HR 1.55 [95% CI 1.21, 1.97]) and CANVAS-R (HR 0.86 [95% CI 0.62, 1.19]) (*p* homogeneity = 0.005), with no increase in fracture risk seen in CANVAS-R, and no reason for the heterogeneity immediately apparent [8].

The goal of these analyses was to explore the effects of canagliflozin compared with placebo on fracture risk in CANVAS compared with CANVAS-R and seek to understand the heterogeneity between the two trials.

## Methods

### CANVAS Program design

The study design, characteristics of participants and the main results of the overall CANVAS Program have previously been published [8–11]. In brief, the CANVAS Program, comprising two similarly designed and conducted trials, CANVAS and CANVAS-R, was designed to assess the cardiovascular and renal safety and efficacy of canagliflozin compared with placebo, and determine how any potential benefits might balance against risks. There were 667 centres in 30 countries in the two trials that were scheduled for joint close-out and analysis when at least 688 cardiovascular events and a minimum of 78 weeks of follow-up had been accrued for the last randomised participant, which occurred in February 2017. The ethics committees at each site approved the protocols for the two trials, and all participants provided written informed consent. All procedures followed were in accordance with the Helsinki Declaration of 1964, as revised in 2013. The trials were registered at ClinicalTrials.gov registration nos. NCT01032629, NCT01989754.

### Participants

Participant inclusion criteria were similar for CANVAS and CANVAS-R. Differences potentially important to fracture occurrence were that rosiglitazone use was an exclusion criterion in CANVAS-R and there was some variation in geographies from which participants were recruited. Participants were those with type 2 diabetes mellitus (glycated haemoglobin ≥53 mmol/mol [7.0%] and ≤ 91 mmol/mol [10.5%]), aged 30 years or older with a history of symptomatic atherosclerotic cardiovascular disease, or 50 years or older with two or more risk factors for cardiovascular disease (duration of diabetes ≥10 years, SBP >140 mmHg while on one or more antihypertensive agents, current smoker, microalbuminuria or macroalbuminuria, or HDL-cholesterol <1 mmol/l). A history of fracture at entry to the trial was based upon reports made by site investigators.

### Randomisation, treatment and follow-up

After a 2 week, single-blind, placebo run-in period, participants were randomised centrally through an interactive web response system using a computer-generated randomisation schedule prepared by the study sponsor using randomly permuted blocks. Participants in CANVAS were assigned in a 1:1:1 ratio to canagliflozin 100 mg, canagliflozin 300 mg or matching placebo, and participants in CANVAS-R were randomly assigned in a 1:1 ratio to canagliflozin or matching placebo, administered at an initial dose of 100 mg once daily with optional up-titration to 300 mg from week 13. Participants and all study staff were masked to individual treatment allocations until the completion of the study. Use of other background therapy for glycaemic management, as well as prevention of cardiovascular outcomes and other diseases was according to best practice instituted in line with local guidelines.

After randomisation, participants had three visits in the first year and were seen at 6 month intervals thereafter, with alternating telephone follow-up between visits. Every follow-up included enquiry about primary and secondary outcome events and adverse events. Fracture events were recorded as part of standard adverse event reporting from the commencement of CANVAS, with more detailed data sought prospectively from 21 March 2014 after the initial report of a possible fracture risk by the DSMC and updating of the case report form. Data describing fracture type, location and other details were sought to support a central review of all fractures by a specialist adjudication committee throughout the entire duration of both CANVAS and CANVAS-R. Core information was recorded by site investigators with copies of imaging reports, diagnostic test data and medical records forwarded in parallel. The updated electronic case report form was in place from the start of CANVAS-R. Falls, regardless of their association with fracture events, were recorded as adverse events identified from spontaneous self-reporting by the participants or from questioning by the investigator at scheduled follow-up. There was no specific diary or other approach implemented to systematically record all falls. Participants who prematurely discontinued study treatment continued scheduled follow-up wherever possible, with extensive efforts made to obtain full outcome data for all serious adverse events, including fracture, until the final follow-up window that spanned from November 2016 to February 2017. Adverse event reporting captured all adverse events (serious and non-serious events) at the initiation of CANVAS but was limited to just serious adverse events and adverse events of special interest (which included fracture) from January 2014, after canagliflozin was registered for marketing.

### Outcomes

The primary outcome of interest for this report was all adjudicated fractures. Additional outcomes evaluated were fractures of different types (low-trauma, high-trauma, pathological, stress and other), fractures at different locations (upper limb, lower limb, spine, pelvis, skull or facial bone or thoracic cage) and fractures that were, and were not, associated with a fall. Intermediate markers related to bone metabolism, including serum calcium, phosphorous, magnesium, alkaline phosphatase (ALP) and body weight, were also evaluated as were effects of canagliflozin on falls (adverse events reported as falls or as a result of falls) and adverse events that might lead to falls, including volume depletion, hypoglycaemia and retinopathy.

### Statistical analysis

All analyses were based on the occurrence of the first event of interest during follow-up. The effect of canagliflozin on fracture risk was assessed in the modified intention-to-treat set (participants who received at least one dose of canagliflozin or placebo) using a Cox proportional hazard model with treatment as the exploratory variable and trial as the stratification factor. Annualised incidence rates per 1000 patient-years of follow-up were calculated for all outcomes in addition to HRs and 95% CIs determined from the model. We tested the homogeneity of treatment effects across the two contributing trials by fitting the interaction term to the model, and the same approach was used for testing comparability of effects across subgroups defined by baseline participant characteristics. Baseline participant characteristics associated with fracture risk were assessed using proportional hazards models. Effects of canagliflozin on intermediate markers related to bone metabolism and body weight were analysed using an ANCOVA model with treatment as an independent effect and adjusting for trial and baseline value. Difference in the least squares means between those assigned to canagliflozin compared with placebo were estimated from the model. For adverse events possibly related to fracture, the HR with 95% CI was estimated from the same form of Cox regression model that was used to determine effects on fracture. Effects on fracture were also estimated according to whether the fracture event occurred on-treatment, within 7, 30 or 90 days of treatment discontinuation or longer after treatment discontinuation. Cumulative event curves were plotted and visually inspected to explore the evolution of fracture risk over time. Analyses were initially done on the integrated CANVAS Program dataset, and then done for CANVAS and CANVAS-R separately. Effects of the 100 mg vs 300 mg doses of canagliflozin were explored in the CANVAS dataset alone. SAS Enterprise Guide version 7.1 (version no. 7.15 HF7 (7.100.5.6177) (64-bit); SAS Institute, Cary, NC, USA) was used for statistical analyses.

## Results

**Fig. 1:**
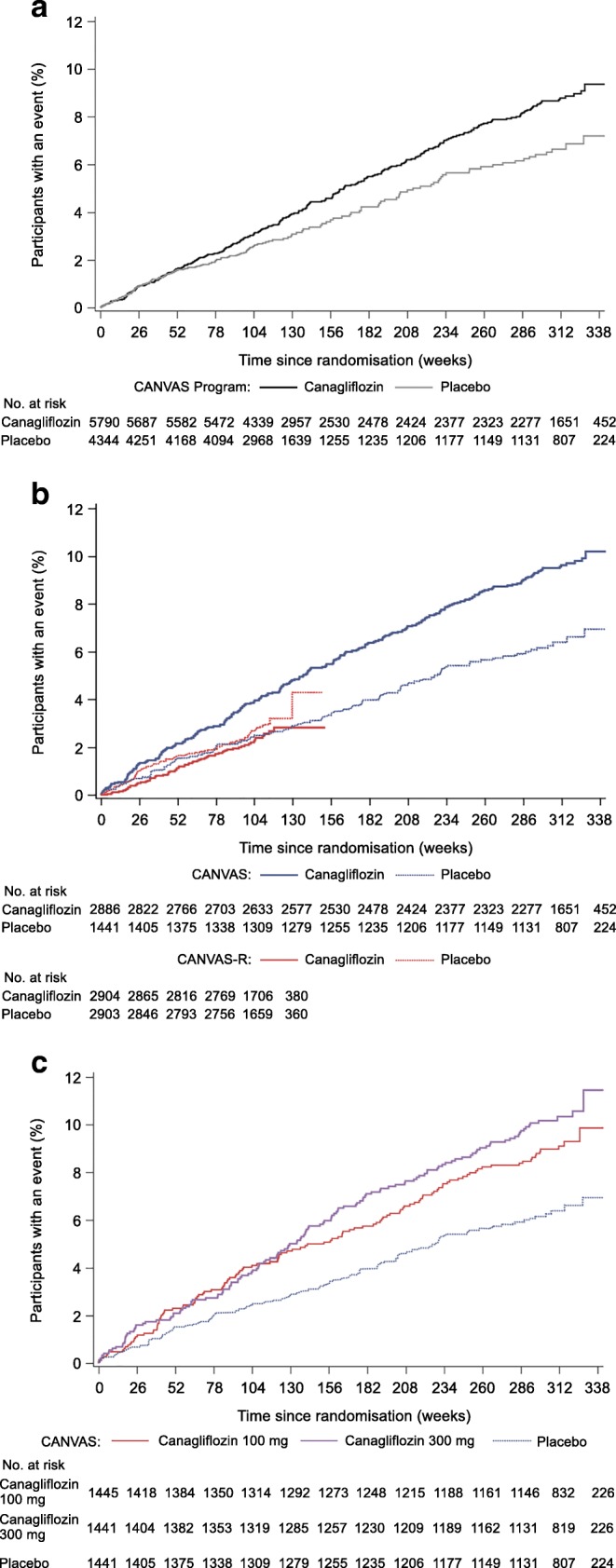
Time to first fracture in (**a**) CANVAS Program, (**b**) CANVAS vs CANVAS-R and (**c**) canagliflozin 100 mg vs 300 mg vs placebo in CANVAS

There were 10,142 participants in the overall CANVAS Program (CANVAS, *n* = 4330 and CANVAS-R, *n* = 5812) and 10,134 who received at least one dose of randomised treatment (electronic supplementary material [ESM] Fig. 1). In CANVAS-R, 73% of participants were up-titrated to the 300 mg dose. The mean follow-up time was 188.2 weeks but varied between the two trials (CANVAS, 295.9 weeks and CANVAS-R, 108.0 weeks). Overall mean age at baseline was 63.3 years, 35.8% were women, mean duration of diabetes was 13.5 years, 65.6% had a history of cardiovascular disease and 21.8% reported a prior history of fracture. There were multiple statistically significant differences between the baseline characteristics of participants included in CANVAS compared with CANVAS-R, but absolute differences between the characteristics of participants in the two trials were mostly small (Table 1).

**Table 1:** Baseline characteristics of participants in CANVAS and CANVAS-R

	CANVAS (*N* = 4327)	CANVAS-R (*N* = 5807)	*p* value (CANVAS vs CANVAS-R)
Age, years	62.4 ± 8.0	64.0 ± 8.3	<0.001^a,^*
Female	1467 (33.9)	2164 (37.3)	<0.001^b,^*
Race			<0.001^b,^*
White	3177 (73.4)	4761 (82.0)	
Asian	795 (18.4)	489 (8.4)	
Black	105 (2.4)	230 (4.0)	
Other^c^	250 (5.8)	327 (5.6)	
Region			<0.001^b,^*
North America	1245 (28.8)	1181 (20.3)	
Central and South America	167 (3.9)	854 (14.7)	
Europe	1335 (30.9)	2271 (39.1)	
ROW	1580 (36.5)	1501 (25.8)	
Current smoker	775 (17.9)	1027 (17.7)	0.77^b^
Hypertension history	3792 (87.6)	5326 (91.7)	<0.001^b,^*
Heart failure history	515 (11.9)	944 (16.3)	<0.001^b,^*
Atrial fibrillation	229 (5.3)	384 (6.6)	0.006^b,^*
Duration of diabetes, years	13.4 ± 7.5	13.7 ± 7.9	0.09^a^
Microvascular disease			
Retinopathy	864 (20.0)	1263 (21.7)	0.03^b,^*
Nephropathy	659 (15.2)	1113 (19.2)	<0.001^b,^*
Neuropathy	1345 (31.1)	1764 (30.4)	0.45^b^
Atherosclerotic vascular disease history^d^			
Coronary	2374 (54.9)	3343 (57.6)	0.007^b,^*
Cerebrovascular	707 (16.3)	1249 (21.5)	<0.001^b,^*
Peripheral	685 (15.8)	1426 (24.6)	<0.001^b,^*
Any	2891 (66.8)	4427 (76.2)	<0.001^b,^*
Cardiovascular disease history^e^	2548 (58.9)	4103 (70.7)	<0.001^b,^*
Fracture history	989 (22.9)	1223 (21.1)	0.03^b,^*
Amputation history	77 (1.8)	160 (2.8)	0.001^b,^*
BMI, kg/m^2^	32.1 ± 6.2	31.9 ± 5.7	0.054^a^
Body weight, kg	91.1 ± 21.3	89.5 ± 19.4	<0.001^a,^*
BP, mmHg			
SBP	136.3 ± 15.7	136.9 ± 15.8	0.046^a,^*
DBP	77.8 ± 9.7	77.6 ± 9.6	0.31^a^
HbA_1c_, mmol/mol	66 ± 9.8	67 ± 9.8	<0.001^a,^*
HbA_1c_, %	8.2 ± 0.9	8.3 ± 0.9	<0.001^a,^*
Cholesterol, mmol/l			
Total	4.4 ± 1.2	4.4 ± 1.2	0.80^a^
HDL	1.2 ± 0.3	1.2 ± 0.3	0.001^a,^*
LDL	2.3 ± 0.9	2.3 ± 0.9	0.56^a^
Ratio of LDL to HDL	2.0 ± 0.9	2.1 ± 0.9	0.10^a^
Triacylglycerol, mmol/l	2.0 ± 1.4	2.1 ± 1.5	<0.001^a,^*
eGFR, ml min^−1^ [1.73 m]^−2^	77.2 ± 18.9	75.9 ± 21.6	0.001^a,^*
Calcium, mmol/l	2.4 ± 0.1	2.4 ± 0.1	0.01^a,^*
Phosphorous, mmol/l	1.2 ± 0.2	1.1 ± 0.2	<0.001^a,^*
Magnesium, mmol/l	0.8 ± 0.1	0.8 ± 0.1	<0.001^a,^*
ALP, U/l	76.3 ± 24.6	76.8 ± 25.4	0.34^a^
Haematocrit, %	41.8 ± 4.2	42.1 ± 4.1	<0.001^a,^*
Median albumin/creatinine ratio (IQR)	11.9 (6.6–36.3)	12.6 (6.7–46.7)	0.13^f^
Normoalbuminuria, no./total no. (%)	3090/4306 (71.8)	3916/5723 (68.4)	<0.001^g,^*
Microalbuminuria, no./total no. (%)	966/4306 (22.4)	1297/5723 (22.7)	
Macroalbuminuria, no./total no. (%)	250/4306 (5.8)	510/5723 (8.9)	
Drug therapy			
Insulin	2172 (50.2)	2921 (50.3)	0.92^b^
Sulfonylurea	2031 (46.9)	2328 (40.1)	<0.001^b,^*
Metformin	3168 (73.2)	4652 (80.1)	<0.001^b,^*
Thiazolidinediones	359 (8.3)	133 (2.3)	<0.001^b,^*
α-Glucosidase inhibitors	117 (2.7)	135 (2.3)	0.23^b^
Glinides	62 (1.4)	0 (0.0)	<0.001^b,^*
DPP-4 inhibitor	317 (7.3)	944 (16.3)	<0.001^b,^*
GLP-1 receptor agonist	96 (2.2)	311 (5.4)	<0.001^b,^*
Statin	3129 (72.3)	4467 (76.9)	<0.001^b,^*
Antithrombotic^h^	3102 (71.7)	4365 (75.2)	<0.001^b,^*
RAAS inhibitor	3488 (80.6)	4625 (79.6)	0.23^b^
β-Blocker	2178 (50.3)	3242 (55.8)	<0.001^b,^*
Diuretics	1900 (43.9)	2589 (44.6)	0.50^b^
Calcium channel blocker	1400 (32.4)	2043 (35.2)	0.003^b,^*

Analyses were performed on data from the on-treatment dataset

Data are mean ± SD or *n* (%), unless otherwise stated

^a^*p* value corresponds to the test for no difference between CANVAS and CANVAS-R from an ANCOVA model

^b^*p* value corresponds to Generalised Cochran–Mantel–Haenszel test for no general association

^c^Includes American Indian or Alaska Native, Native Hawaiian or other Pacific Islander, multiple, other and unknown

^d^Some participants had more than one type of atherosclerotic vascular disease

^e^A history of cardiovascular disease was defined as a history of symptomatic atherosclerotic vascular disease (coronary, cerebrovascular or peripheral)

^f^*p* value corresponds to Wilcoxon rank sum test of equal medians

^g^*p* value corresponds to van Elteren test for no association

^h^Includes antiplatelets and anticoagulants

*p <0.05

IQR, interquartile range; ROW, rest of the world

### Associations of baseline participant characteristics with fracture risk

There were 496 (4.9%) individuals who had a fracture event during follow-up. Participants who had a fracture event during follow-up were different from other trial participants in terms of baseline demography, disease history, laboratory tests, medications used for the management of diabetes and cardiovascular risks. The absolute magnitudes of the differences were small aside from the proportions of women (49.4% vs 35.1%) and the proportions with a prior history of fracture (33.9% vs 21.2%) (ESM Table 1). Univariable modelling identified 20 baseline characteristics that were significantly associated with fracture risk in the overall CANVAS Program (Table 2). The pattern of associations of baseline characteristics with fracture in the univariable analyses were comparable across CANVAS and CANVAS-R except for history of hypertension, serum calcium, haematocrit, albuminuria and antithrombotic use where the associations of these exposures with fracture risk varied between the trials (all *p* heterogeneity <0.047) (Table 2 and ESM Table 2).

**Table 2:** Baseline participant characteristics associated with fracture risk in univariate models in the CANVAS Program, CANVAS and CANVAS-R

	Univariable HR (95% CI)			*p* interaction^a^
	CANVAS Program	CANVAS	CANVAS-R	
Demographics				
Age (1 year higher)	1.04 (1.03, 1.05)*	1.03 (1.02, 1.05)*	1.05 (1.03, 1.07)*	0.30
Female vs male	1.89 (1.58, 2.25)*	2.06 (1.67, 2.54)*	1.53 (1.11, 2.12)*	0.13
Race (white vs non-white)	1.95 (1.51, 2.52)*	2.10 (1.57, 2.81)*	1.53 (0.92, 2.53)	0.28
Race (Asian vs non-Asian)	0.50 (0.37, 0.69)*	0.44 (0.31, 0.63)*	0.89 (0.47, 1.69)	0.06
Region (Europe vs other)	1.34 (1.11, 1.60)*	1.27 (1.02, 1.58)*	1.50 (1.08, 2.08)*	0.40
Region (ROW vs others)	0.64 (0.52, 0.78)*	0.66 (0.52, 0.83)*	0.57 (0.37, 0.88)*	0.56
Disease history				
Hypertension history (Yes vs No)	0.89 (0.68, 1.16)	1.03 (0.75, 1.43)	0.57 (0.35, 0.93)*	0.047*
Atrial fibrillation history (Yes vs No)	1.27 (0.89, 1.81)	1.53 (1.02, 2.30)*	0.82 (0.40, 1.67)	0.14
Duration of diabetes (year greater)	1.04 (1.03, 1.05)*	1.04 (1.02, 1.05)*	1.04 (1.02, 1.05)*	0.94
Retinopathy history (Yes vs No)	1.31 (1.07, 1.60)*	1.15 (0.89, 1.48)	1.72 (1.21, 2.43)*	0.06
Neuropathy history (Yes vs No)	1.21 (1.00, 1.45)*	1.32 (1.07, 1.64)*	0.95 (0.66, 1.36)	0.12
Coronary disease history (Yes vs No)	0.85 (0.71, 1.01)	0.79 (0.64, 0.98)*	0.99 (0.71, 1.37)	0.28
Fracture history (Yes vs No)	1.83 (1.52, 2.20)*	1.63 (1.31, 2.05)*	2.37 (1.69, 3.30)*	0.07
Clinical and laboratory parameters				
DBP (1 mmHg greater)	0.98 (0.97, 0.99)*	0.98 (0.97, 0.99)*	0.98 (0.96, 0.99)*	0.61
HDL-cholesterol (1 mmol/l greater)	1.65 (1.28, 2.13)*	1.76 (1.32, 2.35)*	1.37 (0.83, 2.26)	0.39
LDL-cholesterol (1 mmol/l greater)	0.91 (0.82, 1.00)	0.97 (0.86, 1.08)	0.78 (0.65, 0.95)*	0.06
eGFR (1 ml min^−1^ [1.73 m]^−2^ greater)	0.99 (0.99, 1.00)*	1.00 (0.99, 1.00)	0.99 (0.98, 1.00)*	0.13
Serum calcium (1 mmol/l greater)	0.72 (0.35, 1.49)	1.25 (0.53, 2.92)	0.09 (0.02, 0.45)*	0.005*
ALP (1 U/l greater)	1.00 (1.00, 1.01)*	1.00 (1.00, 1.01)*	1.00 (0.99, 1.01)	0.27
Haematocrit (1% greater)	0.97 (0.95, 0.99)*	0.99 (0.97, 1.02)	0.92 (0.88, 0.96)*	0.002*
Albuminuria (macro or micro vs normal)	1.12 (0.92, 1.35)	0.96 (0.76, 1.22)	1.53 (1.10, 2.13)*	0.03*
Drug therapy				
Insulin (Yes vs No)	1.42 (1.19, 1.70)*	1.39 (1.12, 1.71)*	1.52 (1.09, 2.12)*	0.64
Sulfonylurea (Yes vs No)	0.74 (0.62, 0.89)*	0.77 (0.62, 0.95)*	0.66 (0.47, 0.94)*	0.47
Metformin (Yes vs No)	0.72 (0.59, 0.87)*	0.71 (0.57, 0.89)*	0.74 (0.51, 1.08)	0.83
Statin (Yes vs No)	1.25 (1.01, 1.54)*	1.17 (0.91, 1.49)	1.52 (0.98, 2.36)	0.30
Antithrombotic (Yes vs No)	0.98 (0.81, 1.20)	0.85 (0.68, 1.06)	1.54 (1.00, 2.35)*	0.02*
Diuretics (Yes vs No)	1.35 (1.13, 1.61)*	1.39 (1.13, 1.72)*	1.24 (0.90, 1.72)	0.56
Canagliflozin treatment (Yes vs No)	1.26 (1.04, 1.52)*	1.55 (1.21, 1.97)*	0.86 (0.62, 1.19)	0.005*

^a^*p* interaction between CANVAS and CANVAS-R

*p <0.05

ROW, rest of the world

### Effects of canagliflozin on all fractures, different types of fracture and fractures at different sites

In the overall CANVAS Program, canagliflozin was associated with a higher risk of all fracture compared with placebo, with a rate of 15.40 per 1000 patient-years amongst those assigned canagliflozin, and 11.93 per 1000 patient-years amongst those assigned placebo (Fig. 1). The overall HR was 1.26 (95% CI 1.04, 1.52) but there was significant heterogeneity in the effects on fracture (*p* = 0.005) between the CANVAS trial (HR 1.55 [95% CI 1.21, 1.97]) and the CANVAS-R trial (HR 0.86 [95% CI 0.62, 1.19]) [8]. The cumulative event curves separated at about 12 months for the overall CANVAS Program. For the individual trials, CANVAS showed an immediate separation but for CANVAS-R there was no separation at any time point (Fig. 1 and ESM Table 3). There was no evidence within CANVAS that the risk of fracture associated with the 300 mg dose of canagliflozin was greater than the risk associated with the 100 mg dose (*p* = 0.34).

For the overall CANVAS Program, the point estimates of effect were greater than unity for fractures at all locations and for fractures of all types (except stress fracture and other fracture), though for every one of these fracture subsets the 95% CI crossed unity (Table 3). Different effects on low-trauma fracture and lower limb fracture (both *p* interaction <0.05) drove the difference in overall fracture between the individual trials CANVAS and CANVAS-R, though point estimates of effect for upper limb, pelvis and thoracic cage fractures were also directionally different between the two trials. There was, once again, no significant difference in fracture risk between the 100 mg and 300 mg doses of canagliflozin used in CANVAS for any fracture type or for any fracture location (all *p* >0.26).

**Table 3:** Effects of canagliflozin vs placebo and each dose of canagliflozin vs placebo on fracture types and fracture locations in the CANVAS Program, CANVAS and CANVAS-R

	CANVAS Program HR (95% CI) (canagliflozin vs placebo)	CANVAS HR (95% CI) (canagliflozin vs placebo)	CANVAS-R HR (95% CI) (canagliflozin vs placebo)	*p* interaction^a^	CANVAS		*p* 100 mg vs 300 mg^b^
					HR (95% CI) (100 mg vs placebo)	HR (95% CI) (300 mg vs placebo)	
All fracture	1.26 (1.04, 1.52)^c,^*, *n* = 496	1.55 (1.21, 1.97)^c,^*, *n* = 350	0.86 (0.62, 1.19)^c^, *n* = 146	0.005*	1.45 (1.10, 1.92)*, *n* = 210	1.64 (1.25, 2.15)*,*n* = 225	0.34
Type							
Low-trauma	1.23 (0.99, 1.52)^c^, *n* = 379	1.56 (1.18, 2.06)^c,^*, *n* = 271	0.76 (0.52, 1.12)^c^, *n* = 108	0.003*	1.47 (1.07, 2.01)*, *n* = 162	1.66 (1.22, 2.25)*, *n* = 174	0.39
High-trauma	1.30 (0.83, 2.05), *n* = 89	1.21 (0.71, 2.06), *n* = 66	1.55 (0.67, 3.58), *n* = 23	0.63	1.08 (0.58, 2.00), *n* = 40	1.34 (0.74, 2.42), *n* = 45	0.46
Pathological	1.96 (0.39, 9.91), *n* = 8	1.93 (0.22, 17.30), *n* = 5	2.00 (0.18, 22.00), *n* = 3	0.99	1.93 (0.17, 21.28), *n* = 3	1.94 (0.18, 21.42), *n* = 3	1.00
Stress	0.59 (0.08, 4.27), *n* = 4	0.99 (0.09, 10.90), *n* = 3	–, *n* = 1	–	1.98 (0.18, 21.79), *n* = 3	–, *n* = 1	–
Other^d^	0.82 (0.40, 1.66), *n* = 32	0.72 (0.30, 1.77), *n* = 20	0.99 (0.32, 3.08), *n* = 12	0.67	0.48 (0.15, 1.60), *n* = 12	0.97 (0.36, 2.58), *n* = 16	0.26
Location							
Upper limb^e^	1.25 (0.91, 1.71), *n* = 184	1.40 (0.96, 2.05), *n* = 139	0.95 (0.53, 1.70), *n* = 45	0.28	1.39 (0.90, 2.12), *n* = 87	1.41 (0.92, 2.16), *n* = 88	0.92
Lower limb^f^	1.19 (0.90, 1.58), *n* = 221	1.47 (1.02, 2.11)*, *n* = 156	0.80 (0.49, 1.30), *n* = 65	0.05*	1.35 (0.90, 2.04), *n* = 93	1.58 (1.06, 2.36)*, *n* = 102	0.40
Spine^g^	1.40 (0.72, 2.70), *n* = 43	1.40 (0.62, 3.12), *n* = 31	1.39 (0.44, 4.39), *n* = 12	1.00	1.09 (0.42, 2.83), *n* = 17	1.70 (0.71, 4.06), *n* = 22	0.30
Pelvis	1.36 (0.47, 3.96), *n* = 16	0.85 (0.25, 2.89), *n* = 11	3.96 (0.44, 35.46), *n* = 5	0.23	0.73 (0.16, 3.26), *n* = 7	0.96 (0.24, 3.85), *n* = 8	0.71
Skull or facial bone	1.45 (0.59, 3.57), *n* = 23	1.93 (0.55, 6.86), *n* = 15	1.00 (0.25, 3.98), *n* = 8	0.49	1.93 (0.48, 7.70), *n* = 9	1.94 (0.49, 7.77), *n* = 9	1.00
Thoracic cage	1.34 (0.81, 2.22), *n* = 72	1.85 (0.92, 3.71), *n* = 48	0.84 (0.38, 1.88), *n* = 24	0.15	1.55 (0.70, 3.42), *n* = 26	2.14 (1.02, 4.53)*, *n* = 32	0.33

^a^*p* interaction between CANVAS and CANVAS-R

^b^*p* 100 mg vs 300 mg tests the difference between the canagliflozin 100 mg and 300 mg groups in CANVAS

^c^Previously reported in Neal et al (2017) [8]

^d^Including avascular necrosis, infectious, no trauma or unknown type

^e^Including clavicle, scapula, humerus, radius, ulna, wrist and hand fracture

^f^Including hip, femur, patella, tibia, fibula, ankle, foot and calcaneus fracture

^g^Including cervical, thoracic and lumbar spine fracture

*p <0.05

*n*, number of participants with an event

**Fig. 2:**
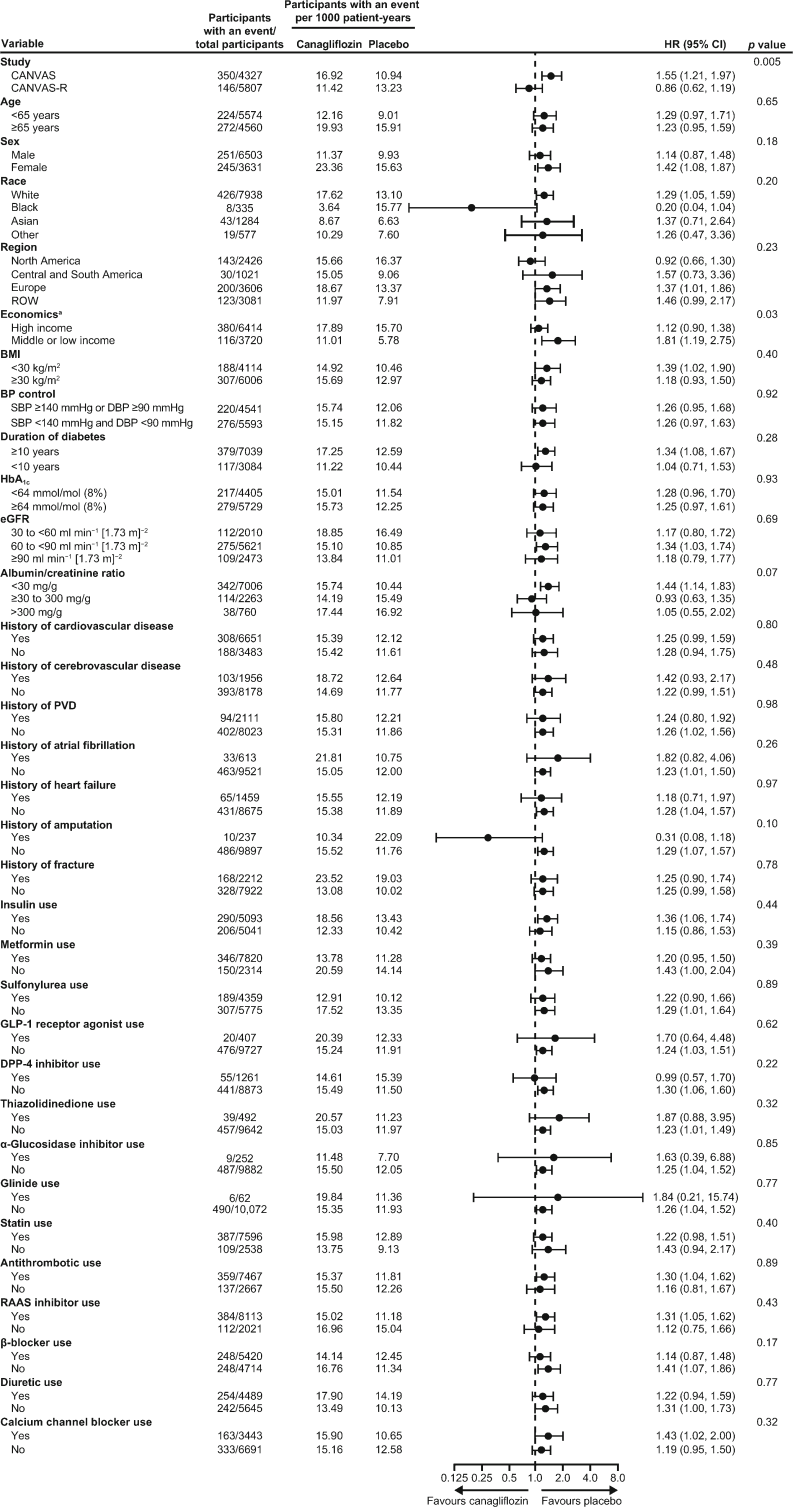
Effects of canagliflozin on fracture in participant subgroups in the CANVAS Program. ^a^According to the 2018 World Bank open data (https://data.worldbank.org/): sites with high economic levels include Australia, Belgium, Canada, Czech Republic, Germany, Spain, Estonia, France, UK, Hungary, Israel, Italy, South Korea, Taiwan, Luxembourg, Netherlands, Norway, New Zealand, Poland, Sweden and USA; sites with low or middle economic levels include Argentina, Brazil, China, Colombia, India, Mexico, Malaysia, Russia and Ukraine. PVD, peripheral vascular disease; ROW, rest of the world

Effects of canagliflozin compared with placebo on fracture risk were also comparable across participant subgroups defined by a broad range of baseline characteristics. The only statistically significant interaction (*p* = 0.03) was for recruitment sites in regions with different levels of economic development (Fig. 2) and there were no interactions by physical characteristics, baseline disease history or use of concomitant medications.

Most participants who sustained a fracture experienced the event while adherent to double-blind randomised treatment (419/496) (ESM Table 4). The HR for fracture with canagliflozin compared with placebo did not differ when analyses included only participants who experienced fracture while using randomised treatment or shortly after discontinuing randomised treatment.

### Effects of canagliflozin on falls and adverse events that might lead to falls

Canagliflozin use was associated with an increased risk of adverse events or serious adverse events attributed to falls in CANVAS, with a similar effect observed for adverse events related to volume depletion. No corresponding increases in falls reported as serious adverse events was observed in CANVAS-R and in neither trial was there a clear effect on hypoglycaemia or retinopathy. There was evidence in CANVAS that falls occurred at greater rates with the 300 mg compared with 100 mg dose of canagliflozin (*p* = 0.01) (Table 4). Across the overall CANVAS Program, there were 200 fractures identified as associated with a fall, 52 that were identified as not associated with a fall and 267 for which the relationship with a fall was uncertain. An adverse effect of canagliflozin on fracture risk in CANVAS was apparent for fractures with an unclear association with fall.

**Table 4:** Effects of canagliflozin vs placebo and each dose of canagliflozin vs placebo on fractures related and unrelated to falls, adverse events attributed to falls or possibly causing falls and biomarkers of bone metabolism in the CANVAS Program, CANVAS and CANVAS-R

	CANVAS Program (canagliflozin vs placebo)	CANVAS (canagliflozin vs placebo)	CANVAS-R (canagliflozin vs placebo)	*p* inter- action^a^	CANVAS		*p* 100 mg vs 300 mg^b^
					(100 mg vs placebo)	(300 mg vs placebo)	
Fractures related and unrelated to falls, HR (95% CI)							
Fall associated^c^	–	0.99 (0.63, 1.56), *n* = 86	0.75 (0.51, 1.08), *n* = 114	–	1.06 (0.64, 1.77), *n* = 59	0.92 (0.54, 1.57), *n* = 55	0.58
Non-fall associated^c^	–	0.69 (0.26, 1.81), *n* = 17	1.69 (0.85, 3.35), *n* = 35	–	0.82 (0.28, 2.45), *n* = 13	0.55 (0.16, 1.89), *n* = 11	0.53
Unclear^c^	–	1.79 (1.34, 2.39)*, *n* = 267	–	–	1.59 (1.14, 2.21)*, *n* = 151	1.99 (1.45, 2.73)*, *n* = 174	0.10
Adverse events^d^, HR (95% CI)							
Falls^e^	–	1.59 (1.02, 2.49)*, *n* = 108	0.88 (0.36, 2.18), *n* = 19	–	1.13 (0.67, 1.93), *n* = 55	2.06 (1.28, 3.31)*, *n* = 78	0.01*
Volume depletion	–	1.44 (1.09, 1.90)*, *n* = 266	1.02 (0.61, 1.70), *n* = 59	–	1.28 (0.93, 1.76), *n* = 157	1.61 (1.19, 2.18)*, *n* = 176	0.12
Hypoglycaemia	–	1.13 (0.94, 1.35), *n* = 551	1.26 (0.68, 2.34), *n* = 41	–	1.03 (0.83, 1.27), *n* = 346	1.23 (1.00, 1.51)*, *n* = 373	0.09
Retinopathy	–	1.16 (0.86, 1.56), *n* = 210	1.38 (0.44, 4.35), *n* = 12	–	1.04 (0.74, 1.47), *n* = 129	1.27 (0.91, 1.78), *n* = 142	0.23
Biomarkers of bone metabolism and body weight, mean difference between treatment groups during follow-up (95% CI)^f^							
Serum calcium (mmol/l)	0.006 (0.002, 0.010)*	0.015 (0.008, 0.021)*	0.0001 (−0.005, 0.005)	<0.001*	0.015 (0.008, 0.023)*	0.014 (0.006, 0.022)*	0.72
Serum phosphorous (mmol/l)	−0.007 (−0.013, −0.0001)*	0.008 (−0.003, 0.018)	−0.016 (−0.025, −0.008)*	<0.001*	0.006 (−0.006, 0.018)	0.009 (−0.003, 0.022)	0.58
Serum magnesium (mmol/l)	0.033 (0.029, 0.036)*	0.062 (0.056, 0.067)*	0.013 (0.009, 0.017)*	<0.001*	0.054 (0.047, 0.060)*	0.070 (0.063, 0.076)*	<0.001*
ALP (U/l)	0.264 (−1.181, 1.709)	−0.917 (−3.227, 1.392)	1.010 (−0.840, 2.861)	0.19	−1.450 (−3.671, 0.771)	−0.381 (−3.308, 2.546)	0.46
Body weight (kg)	−2.403 (−2.639, −2.167)*	−2.545 (−2.988, −2.103)*	−2.309 (−2.566, −2.053)*	0.33	−2.301 (−2.809, −1.793)*	−2.796 (−3.307, −2.285)*	0.06

^a^*p* interaction between CANVAS and CANVAS-R

^b^*p* 100 mg vs 300 mg test difference between 100 mg canagliflozin and 300 mg canagliflozin groups in CANVAS

^c^The information on whether a fracture was associated with a fall was recorded since March 2014 in CANVAS. It was recorded from the beginning of CANVAS-R

^d^Analyses were performed on data from the on-treatment dataset (participants who had a safety outcome while they were receiving canagliflozin or placebo or within 30 days after discontinuation of the drug or placebo). In CANVAS, these adverse events up until 7 January 2014 were included for analysis, because after this time, only serious adverse events or adverse events leading to discontinuation were collected. In CANVAS-R, only serious adverse events or adverse events leading to discontinuation were collected. Owing to the differences between the two trials in methods of collection of the data, an integrated analysis of these adverse events is not possible

^e^Adverse events reported as falls or as a result of falls

^f^The mean treatment difference of canagliflozin compared with placebo (from baseline to the last measurement of the trial) in the least squares means and associated 95% CIs were estimated from an ANCOVA model with treatment as an independent effect and adjusting for trial and baseline value

*p <0.05

*n*, number of participants with an event

### Effects of canagliflozin on biomarkers of bone metabolism

Effects of canagliflozin on serum calcium, serum phosphorous and serum magnesium differed between CANVAS and CANVAS-R (all *p* interaction <0.001) though mean levels remained within normal ranges throughout the studies (Table 4). Serum calcium was increased in CANVAS but not CANVAS-R; phosphorous was decreased in CANVAS-R but not CANVAS; and magnesium was increased in both studies, but to a greater extent in CANVAS and especially in the 300 mg dose group. ALP was unchanged in all analyses and body weight was decreased consistently with no heterogeneity by trial and no difference by dose in CANVAS (*p* = 0.06). Effects of canagliflozin compared with placebo on calcium, phosphorous and magnesium did not differ between CANVAS and CANVAS-R in analyses restricted to assays done while participants were using randomised treatment (ESM Table 5). Likewise, differences between studies were attenuated in analyses that compared biomarker levels between canagliflozin and placebo at the same follow-up time in both trials (130 weeks).

## Discussion

We could find no definitive explanation for either the fracture risk observed in the overall CANVAS Program or for the difference in fracture risk between CANVAS and CANVAS-R. In practice, however, most fractures are the result of a fall [15] and, in the absence of a clear signal for metabolic bone disease, this remains the most plausible mechanism by which a real effect on fracture in CANVAS would be mediated. While the analyses themselves identified no direct evidence for an association between falls and fracture risk, the available data to assess such an association were limited and a fall-related mechanism of action may have been missed. The chief alternative possibility is that the observed increase in fracture risk in the CANVAS Program is a chance finding. The likelihood of chance being the explanation is amplified by the absence of any identified mechanism of action, the initial identification of the fracture risk during serial six monthly data and safety reviews and the inconsistency of the findings for fracture across the two constituent trials. The absence of a fracture risk in the recently reported CREDENCE (Canagliflozin and Renal Events in Diabetes with Established Nephropathy Clinical Evaluation) trial [16] of canagliflozin amongst participants with type 2 diabetes and chronic kidney disease increases the likelihood of chance being the correct interpretation of the outlying CANVAS result. Alternatively, if the fracture risk is real, then use of the 100 mg dosing strategy employed in CANVAS-R and CREDENCE might have ameliorated the risk of fracture, though further investigation would be required to confirm this.

The other large completed trials of an SGLT2 inhibitor, the EMPA-REG OUTCOME trial and the DECLARE (Dapagliflozin Effect on Cardiovascular Events)–TIMI 58 trial, identified no risk of fracture [17–19], and the labelled fracture risk for dapagliflozin [14] relates only to individuals with moderate renal impairment and is based on a very small number of events. A meta-analysis of all available fracture data for SGLT2 inhibitors did not identify an overall risk of fracture, but there was some evidence of heterogeneity between compounds that was driven substantively by the data from CANVAS [20]. Observational studies of SGLT2 inhibitor use and fracture risk have identified no clear increase in risk [21, 22].

Our detailed analyses of the CANVAS Program identified multiple and mostly expected observational associations of baseline patient characteristics with the risk of subsequent fracture. Placebo group rates of fracture in CANVAS-R were marginally higher than in CANVAS, but these rate differences provide no explanation for the different effects of the drug across the two studies. Where unanticipated associations were observed, or where there were differences in the associations of risk factors with fracture between CANVAS and CANVAS-R, the differences were small and there was no discernible pattern or identifiable mechanistic pathway that was obviously linked to the causation of fracture. The multiple analyses performed increased the likelihood of chance significant findings, and this may be the explanation for some or all of these observations. Extensive investigation of baseline characteristics as possible modifiers of the effect of canagliflozin on fracture risk using subgroup analyses, likewise, could identify no reason for either the association of canagliflozin with fracture risk in the overall CANVAS Program or explain the differences in effects between CANVAS and CANVAS-R. While statistical power to detect interactions with randomised treatment was only moderate, and these analyses could be affected by the heterogeneity between the two trials, strong effects able to explain the large difference in fracture risk between trials are unlikely to have been missed.

An increased risk of falling caused by complications of diabetes such as retinopathy or neuropathy, or side effects of glucose-lowering agents such as hypoglycaemia or hypovolaemia, is a plausible mechanism for the reported increased risk of fracture amongst individuals with type 2 diabetes [23, 24]. It has also been proposed as an explanation for reported greater fracture risks in sites that are less economically developed. These analyses did not identify direct evidence of an increased risk of fall-related fracture, but the data are limited because adverse event reporting typically focuses on the outcome of events rather than the mechanism leading to the event. There was no systematic recording of all falls during the trials, and the systematic collection of information about falls occurring at the time of fracture was only implemented after the initial signal for fracture risk was identified in 2013. Of all the fracture events analysed, information about the presence or absence of a concurrent fall was available for less than half because retrospective data collection was not able to reliably elucidate this information for historical events.

Although data on falls in general or falls reported as adverse events was not systematically collected throughout the duration of the CANVAS Program, the data suggest that canagliflozin treatment may increase the risk of falls. Canagliflozin was also associated with an increased risk of volume depletion events, findings which provide some indirect support for a fall-related mechanism driving the increase in fracture risk. The differences in the effects of canagliflozin on the risk of falls and volume depletion events in CANVAS compared with CANVAS-R align with the different effects on fracture between the two trials. However, these data require cautious interpretation—while the null effect on falls and volume depletion events in CANVAS-R could be real, perhaps due to a more cautious up-titration of dose in CANVAS-R amongst investigators sensitised to the risk of fracture, it is also possible that the collection of only serious adverse events in CANVAS-R reduced the capacity to detect effects on falls and volume depletion events, which were frequently categorised as non-serious. In addition, the possibility that physicians may have been able to ameliorate fracture risk by avoiding the higher dose or concomitant BP-lowering therapies amongst those with the potential for harm presupposes that the 300 mg dose of canagliflozin is associated with a greater risk than the 100 mg dose. A dose response effect was observed for the effect on falls but not for other adverse events possibly related to fall risk, or fracture risk itself. Moreover, the 100 mg dose of canagliflozin was associated with an increased risk of fracture, but there were no detectable effects of the 100 mg dose on either fall events or volume depletion events. The likelihood that the different dosing regimens explains the different effects of canagliflozin on fracture between CANVAS and CANVAS-R is further reduced by the observation that the event curves for the canagliflozin 100 mg and 300 mg doses were overlapping until about 2.5 years into follow-up. The different follow-up durations in CANVAS compared with CANVAS-R also fail to provide an explanation for the differences in the effects observed between the two trials, with adverse effects clearly apparent early in CANVAS but not in CANVAS-R.

Detailed markers of bone metabolism were not measured in the CANVAS Program but there were effects of canagliflozin compared with placebo on mean levels of serum calcium, phosphorous and magnesium, and these differed between CANVAS and CANVAS-R. The absolute magnitude of all effects on these indicators was small and inconsistent, and unlikely to be of clinical significance. There is no identified mechanism by which the observed changes would explain the overall effect on fracture or the observed heterogeneity in the risk of fracture between CANVAS and CANVAS-R. In part, the differences in the effects on these biomarkers between the two trials may be a result of the different durations of follow-up, since heterogeneity was much reduced when analyses were done at comparable follow-up times when mean adherence to treatment was more comparable. However, the fact that the increased risk of fracture was observed in CANVAS almost immediately and not observed in CANVAS-R over 2.5 years suggests that any time-dependent effects on calcium, phosphorous or magnesium, if real, do not explain the heterogeneity in fracture risk between CANVAS and CANVAS-R. In the absence of external data describing substantive adverse effects of canagliflozin [25, 26], or other SGLT2 inhibitors, on markers of bone turnover, a pathological mechanism based on bone metabolism effects seems an unlikely cause of the fractures observed in CANVAS. Sex is an important determinant of fracture risk, and while not identified as a factor likely to explain the different risks observed in CANVAS vs CANVAS-R, the under-representation of women in the trials reduced the power to assess the effects of sex and represents an ongoing challenge to trialists.

In conclusion, we could find no clear explanation for either the fracture risk observed in the CANVAS Program or the difference in fracture risk observed in CANVAS compared with CANVAS-R. In practice, however, most fractures are caused by falls and the observed association is likely a consequence of either an unidentified fall-related mechanism or else a chance finding. The null finding for fracture risk with canagliflozin in the recently completed CREDENCE trial has provided important additional insight and somewhat increases the likelihood that the adverse effect observed in CANVAS was a spurious finding.

## Electronic supplementary material

ESM (PDF 283 kb)

## Data Availability

Data from the overall CANVAS Program will be made available in the public domain via the Yale University Open Data Access Project (YODA; http://yoda.yale.edu/) once the product and relevant indication have been approved by regulators in the USA and European Union and the study has been completed for 18 months.

## Abbreviations

ALP: Alkaline phosphatase
CANVAS: CANagliflozin cardioVascular Assessment Study
CANVAS-R: CANagliflozin cardioVascular Assessment Study-Renal
CREDENCE: Canagliflozin and Renal Events in Diabetes with Established Nephropathy Clinical Evaluation
DBP: Diastolic BP
DPP-4: Dipeptidyl peptidase-4
DSMC: Data and Safety Monitoring Committee
GLP-1: Glucagon-like peptide-1
IQR: Interquartile range
PVD: Peripheral vascular disease
RAAS: Renin–angiotensin–aldosterone system
SBP: Systolic BP
SGLT2: Sodium glucose co-transporter 2
