# Supplementary material for: Canagliflozin and fracture risk in individuals with type 2 diabetes: results from the CANVAS Program
Source: Diabetologia. 2019 Aug 10;62(10):1854–67. doi: 10.1007/s00125-019-4955-5 (PMC6731200; doi:10.1007/s00125-019-4955-5)
Supplement: Supplementary file 1 — (PDF 283 kb) [file 125_2019_4955_MOESM1_ESM.pdf]

ELECTRONIC SUPPLEMENTARY MATERIAL (ESM)

ESM Table 1. Baseline characteristics of the participants with and without fracture during follow-up in the CANVAS Program

	Participants with fracture (n=496)	Participants without fracture (n=9638)	p value (with vs. without)
Study — no. (%)			<0.001[a],*
CANVAS	350 (70.6)	3977 (41.3)	
CANVAS-R	146 (29.4)	5661 (58.7)	
Age — year, mean±SD	65.2±8.1	63.2±8.2	<0.001[b],*
Female — no. (%)	245 (49.4)	3386 (35.1)	<0.001[a],*
Race — no. (%)			<0.001[a],*
White	426 (85.9)	7512 (77.9)	
Asian	43 (8.7)	1241 (12.9)	
Black	8 (1.6)	327 (3.4)	
Other[c]	19 (3.8)	558 (5.8)	
Region — no. (%)			<0.001[a],*
North America	143 (28.8)	2283 (23.7)	
Central and South America	30 (6.0)	991 (10.3)	
Europe	200 (40.3)	3406 (35.3)	
Rest of the world	123 (24.8)	2958 (30.7)	
Current smoker — no. (%)	79 (15.9)	1723 (17.9)	0.27[a]
Hypertension history — no. (%)	435 (87.7)	8683 (90.1)	0.08[a]
Heart failure history— no. (%)	65 (13.1)	1394 (14.5)	0.40[a]
Atrial fibrillation—no. (%)	33 (6.7)	580 (6.0)	0.56[a]
Duration of diabetes —year, mean±SD	15.7±8.4	13.4±7.7	<0.001[b],*
Microvascular disease — no. (%)			
Retinopathy	124 (25.0)	2003 (20.8)	0.02[a],*
Nephropathy	90 (18.1)	1682 (17.5)	0.69[a]
Neuropathy	171 (34.5)	2938 (30.5)	0.06[a]
Atherosclerotic vascular disease history — no. (%)[d]			

Coronary	254 (51.2)	5463 (56.7)	0.02[a,*]
Cerebrovascular	103 (20.8)	1853 (19.2)	0.40[a]
Peripheral	94 (19.0)	2017 (20.9)	0.29[a]
Any	341 (68.8)	6977 (72.4)	0.08[a]
Cardiovascular disease history – no. (%)[e]	308 (62.1)	6343 (65.8)	0.09[a]
Fracture history — no. (%)	168 (33.9)	2044 (21.2)	<0.001[a,*]
Amputation history — no. (%)	10 (2.0)	227 (2.4)	0.63[a]
Body mass index – kg/m^2, mean±SD	32.3±6.4	31.9±5.9	0.16[b]
Body weight — kg, mean±SD	90.1±20.9	90.2±20.2	0.97[b]
Blood pressure — mmHg, mean±SD			
Systolic blood pressure	136.5±16.5	136.6±15.7	0.83[b]
Diastolic blood pressure	75.9±9.9	77.8±9.6	<0.001[b,*]
HbA_{1c}— mmol/mol, mean±SD	66±9.8	67±9.8	0.19[b]
HbA_{1c} — %, mean±SD	8.2±0.9	8.3±0.9	0.19[b]
Cholesterol — mmol/l, mean±SD			
Total	4.3±1.2	4.4±1.2	0.54[b]
HDL	1.2±0.3	1.2±0.3	<0.001[b,*]
LDL	2.2±0.9	2.3±0.9	0.04[b,*]
Ratio of LDL to HDL	1.9±1.0	2.0±0.9	<0.001[b,*]
Triglycerides — mmol/l, mean±SD	2.0±1.9	2.0±1.4	0.98[b]
eGFR — ml min^{-1} [1.73 m]$^{-2}$, mean±SD	74.5±20.2	76.6±20.5	0.03[b,*]
Calcium — mmol/l, mean±SD	2.4±0.1	2.4±0.1	0.18[b]
Phosphorous — mmol/l, mean±SD	1.2±0.2	1.2±0.2	0.02[b,*]
Magnesium — mmol/l, mean±SD	0.8±0.1	0.8±0.1	0.49[b]
ALP — U/L, mean±SD	78.5±24.4	76.5±25.1	0.08[b]
Haematocrit — %, mean±SD	41.4±4.2	42.0±4.1	0.002[b,*]
Median albumin:creatinine ratio (IQR)	13.0 (7.2-43.8)	12.2 (6.6-42.0)	0.41[f]
Normoalbuminuria —no./total no. (%)	342/494 (69.2)	6664/9535 (69.9)	0.76[g]
Microalbuminuria —no./total no. (%)	114/494 (23.1)	2149/9535 (22.5)	
Macroalbuminuria —no./total no. (%)	38/494 (7.7)	722/9535 (7.6)	
Drug therapy — no. (%)			
Insulin	290 (58.5)	4803 (49.8)	<0.001[a,*]

Sulphonylurea	189 (38.1)	4170 (43.3)	0.02[a,*]
Metformin	346 (69.8)	7474 (77.5)	<0.001[a,*]
Thiazolidinediones	39 (7.9)	453 (4.7)	0.001[a,*]
α-glucosidase inhibitors	9 (1.8)	243 (2.5)	0.32[a]
Glinides	6 (1.2)	56 (0.6)	0.08[a]
DPP-4 inhibitor	55 (11.1)	1206 (12.5)	0.35[a]
GLP-1 receptor agonist	20 (4.0)	387 (4.0)	0.99[a]
Statin	387 (78.0)	7209 (74.8)	0.11[a]
Antithrombotic[h]	359 (72.4)	7108 (73.7)	0.50[a]
RAAS inhibitor	384 (77.4)	7729 (80.2)	0.13[a]
β-blocker	248 (50.0)	5172 (53.7)	0.11[a]
Diuretics	254 (51.2)	4235 (43.9)	0.002[a,*]
Calcium channel blocker	163 (32.9)	3280 (34.0)	0.59[a]

Abbreviations: ALP, alkaline phosphatase; CANVAS, CANagliflozin cardioVascular Assessment Study; CANVAS-R, CANagliflozin cardioVascular Assessment Study–Renal; DPP-4, dipeptidyl peptidase-4; eGFR, estimated glomerular filtration rate; GLP-1, glucagon-like peptide-1; HDL, high-density lipoprotein; IQR, interquartile range; LDL, low-density lipoprotein; RAAS, renin-angiotensin-aldosterone system; SD, standard deviation.

[a]p value corresponds to Generalised Cochran-Mantel-Haenszel test for no general association.

[b]p value corresponds to the test for no difference between those with and without fracture during follow-up from analysis of variance (ANCOVA) model.

[c]Includes American Indian or Alaska Native, Native Hawaiian or other Pacific Islander, multiple, other, and unknown.

[d]Some patients had more than one type of atherosclerotic vascular disease.

[e]A history of cardiovascular disease was defined as a history of symptomatic atherosclerotic vascular disease (coronary, cerebrovascular, or peripheral).

[f]p value corresponds to Wilcoxon rank sum test of equal medians.

[g]p value corresponds to Van Elteren test for no association.

[h]Includes antiplatelets and anticoagulants.

*$p < 0.05$.

ESM Table 2. Association of baseline participant characteristics with risk of fracture in a univariate model in the CANVAS Program, CANVAS, and CANVAS-R

	Univariable HR (95% CI)			p interaction[a]
	CANVAS Program	CANVAS	CANVAS-R	
**Demographics**				
Age (1 year higher)	1.04 (1.03, 1.05)*	1.03 (1.02, 1.05)*	1.05 (1.03, 1.07)*	0.30
Female vs. male	1.89 (1.58, 2.25)*	2.06 (1.67, 2.54)*	1.53 (1.11, 2.12)*	0.13
Race				
White vs. non-white	1.95 (1.51, 2.52)*	2.10 (1.57, 2.81)*	1.53 (0.92, 2.53)	0.28
Asian vs. non-Asian	0.50 (0.37, 0.69)*	0.44 (0.31, 0.63)*	0.89 (0.47, 1.69)	0.06
Black vs. non-Black	0.62 (0.31, 1.25)	0.66 (0.27, 1.60)	0.56 (0.18, 1.75)	0.82
Region				
North America vs. others	1.16 (0.95, 1.41)	1.17 (0.93, 1.47)	1.12 (0.76, 1.65)	0.86
Central/South America vs. others	0.93 (0.64, 1.35)	1.15 (0.69, 1.94)	0.76 (0.45, 1.30)	0.28
Europe vs. other	1.34 (1.11, 1.60)*	1.27 (1.02, 1.58)*	1.50 (1.08, 2.08)*	0.40
ROW vs. others	0.64 (0.52, 0.78)*	0.66 (0.52, 0.83)*	0.57 (0.37, 0.88)*	0.56
Current smoker (Yes vs. No)	0.88 (0.69, 1.12)	0.89 (0.67, 1.18)	0.85 (0.55, 1.33)	0.88
Hypertension history (Yes vs. No)	0.89 (0.68, 1.16)	1.03 (0.75, 1.43)	0.57 (0.35, 0.93)*	0.047*
Heart failure history (Yes vs. No)	1.03 (0.79, 1.33)	1.21 (0.89, 1.65)	0.72 (0.44, 1.18)	0.08
Atrial fibrillation history (Yes vs. No)	1.27 (0.89, 1.81)	1.53 (1.02, 2.30)*	0.82 (0.40, 1.67)	0.14
Duration of diabetes (year greater)	1.04 (1.03, 1.05)*	1.04 (1.02, 1.05)*	1.04 (1.02, 1.05)*	0.94
Microvascular disease history				
Retinopathy (Yes vs. No)	1.31 (1.07, 1.60)*	1.15 (0.89, 1.48)	1.72 (1.21, 2.43)*	0.06
Nephropathy (Yes vs. No)	1.14 (0.91, 1.44)	1.18 (0.89, 1.55)	1.07 (0.71, 1.61)	0.71
Neuropathy (Yes vs. No)	1.21 (1.00, 1.45)*	1.32 (1.07, 1.64)*	0.95 (0.66, 1.36)	0.12
Vascular disease history				
Coronary (Yes vs. No)	0.85 (0.71, 1.01)	0.79 (0.64, 0.98)*	0.99 (0.71, 1.37)	0.28
Cerebrovascular (Yes vs. No)	1.22 (0.99, 1.52)	1.28 (0.99, 1.67)	1.11 (0.76, 1.63)	0.55
Peripheral (Yes vs. No)	1.06 (0.84, 1.32)	1.09 (0.82, 1.44)	1.00 (0.68, 1.46)	0.72
Any (Yes vs. No)	0.99 (0.82, 1.19)	0.91 (0.73, 1.13)	1.27 (0.85, 1.90)	0.15
Cardiovascular disease history (Yes vs. No)	1.03 (0.86, 1.24)	0.97 (0.79, 1.20)	1.21 (0.83, 1.74)	0.33

Fracture history (Yes vs. No)	1.83 (1.52, 2.20)*	1.63 (1.31, 2.05)*	2.37 (1.69, 3.30)*	0.07
Amputation history (Yes vs. No)	1.02 (0.54, 1.90)	1.17 (0.55, 2.47)	0.78 (0.25, 2.44)	0.56
**Clinical and laboratory parameters**				
Body mass index (1 unit greater)	1.01 (0.99, 1.02)	1.02 (1.00, 1.03)	0.99 (0.96, 1.02)	0.17
Systolic blood pressure (1 mmHg greater)	1.00 (0.99, 1.01)	1.00 (1.00, 1.01)	1.00 (0.99, 1.01)	0.54
Diastolic blood pressure (1 mmHg greater)	0.98 (0.97, 0.99)*	0.98 (0.97, 0.99)*	0.98 (0.96, 0.99)*	0.61
HbA_{1c} (10.9 mmol/mol [1%] greater)	0.98 (0.89, 1.08)	1.00 (0.89, 1.12)	0.95 (0.80, 1.13)	0.62
Total cholesterol (1 mmol/l greater)	0.99 (0.91, 1.07)	1.03 (0.95, 1.13)	0.88 (0.75, 1.02)	0.07
HDL cholesterol (1 mmol/l greater)	1.65 (1.28, 2.13)*	1.76 (1.32, 2.35)*	1.37 (0.83, 2.26)	0.39
LDL cholesterol (1 mmol /l greater)	0.91 (0.82, 1.00)	0.97 (0.86, 1.08)	0.78 (0.65, 0.95)*	0.06
Triglycerides (1 mmol/l greater)	1.02 (0.96, 1.08)	1.02 (0.95, 1.10)	1.02 (0.92, 1.14)	1.00
eGFR (1 ml min^{-1} $[1.73\ m]^{-2}$ greater)	0.99 (0.99, 1.00)*	1.00 (0.99, 1.00)	0.99 (0.98, 1.00)*	0.13
Serum calcium (1 mmol/l greater)	0.72 (0.35, 1.49)	1.25 (0.53, 2.92)	0.09 (0.02, 0.45)*	0.005*
Serum phosphorous (1 mmol/l greater)	1.55 (0.95, 2.54)	1.35 (0.75, 2.43)	2.17 (0.88, 5.36)	0.39
Serum magnesium (1 mmol/l greater)	0.72 (0.27, 1.89)	0.91 (0.28, 2.94)	0.42 (0.07, 2.35)	0.46
Alkaline phosphatase (1 U/l greater)	1.00 (1.00, 1.01)*	1.00 (1.00, 1.01)*	1.00 (0.99, 1.01)	0.27
Haematocrit (1% greater)	0.97 (0.95, 0.99)*	0.99 (0.97, 1.02)	0.92 (0.88, 0.96)*	0.002*
Albuminuria (macro or micro vs. normal)	1.12 (0.92, 1.35)	0.96 (0.76, 1.22)	1.53 (1.10, 2.13)*	0.03*
**Drug therapy**				
Insulin (Yes vs. No)	1.42 (1.19, 1.70)*	1.39 (1.12, 1.71)*	1.52 (1.09, 2.12)*	0.64
Sulphonylurea (Yes vs. No)	0.74 (0.62, 0.89)*	0.77 (0.62, 0.95)*	0.66 (0.47, 0.94)*	0.47
Metformin (Yes vs. No)	0.72 (0.59, 0.87)*	0.71 (0.57, 0.89)*	0.74 (0.51, 1.08)	0.83
Thiazolidinediones (Yes vs. No)	1.20 (0.86, 1.66)	1.16 (0.81, 1.65)	1.51 (0.62, 3.69)	0.58
α-glucosidase inhibitors (Yes vs. No)	0.69 (0.36, 1.34)	0.60 (0.27, 1.35)	0.97 (0.31, 3.03)	0.51
Glinides (Yes vs. No)	1.18 (0.53, 2.64)	1.18 (0.53, 2.64)	-	-
DPP-4 inhibitor (Yes vs. No)	1.12 (0.84, 1.48)	0.88 (0.57, 1.34)	1.43 (0.97, 2.12)	0.10
GLP-1 receptor agonist (Yes vs. No)	1.28 (0.82, 2.00)	1.03 (0.51, 2.07)	1.54 (0.85, 2.78)	0.38
Statin (Yes vs. No)	1.25 (1.01, 1.54)*	1.17 (0.91, 1.49)	1.52 (0.98, 2.36)	0.30
Antithrombotic (Yes vs. No)	0.98 (0.81, 1.20)	0.85 (0.68, 1.06)	1.54 (1.00, 2.35)*	0.02*
RAAS inhibitor (Yes vs. No)	0.83 (0.67, 1.03)	0.88 (0.68, 1.13)	0.74 (0.51, 1.07)	0.47
β-blocker (Yes vs. No)	0.92 (0.78, 1.10)	0.83 (0.67, 1.02)	1.20 (0.86, 1.68)	0.06
Diuretics (Yes vs. No)	1.35 (1.13, 1.61)*	1.39 (1.13, 1.72)*	1.24 (0.90, 1.72)	0.56

Calcium channel blocker (Yes vs. No)	0.98 (0.81, 1.19)	1.05 (0.84, 1.30)	0.85 (0.60, 1.20)	0.33
Canagliflozin treatment (Yes vs. No)	1.26 (1.04, 1.52)*	1.55 (1.21, 1.97)*	0.86 (0.62, 1.19)	0.005*

Abbreviations: CANVAS, CANagliflozin cardioVascular Assessment Study; CANVAS-R, CANagliflozin cardioVascular Assessment Study–Renal; CI, confidence interval; DPP-4, dipeptidyl peptidase-4; eGFR, estimated glomerular filtration rate; GLP-1, glucagon-like peptide-1; HDL, high-density lipoprotein; HR, hazard ratio; LDL, low-density lipoprotein; RAAS, renin-angiotensin-aldosterone system; ROW, rest of the world.

[a]p interaction between CANVAS and CANVAS-R.

*$p < 0.05$.

ESM Table 3. Effects of canagliflozin versus placebo on fracture risk early, midway, and late during follow-up in the CANVAS Program, CANVAS, and CANVAS-R

	Participants with an event per 1000 patient-years		
	**Canagliflozin**	**Placebo**	**HR (95% CI)**
**CANVAS Program**			
≤1 year	16.34	15.75	0.98 (0.71, 1.34)
>1 year and ≤2 years	15.69	10.37	1.45 (0.99, 2.11)
>2 years and ≤4 years	17.32	12.98	1.30 (0.90, 1.88)
>4 years	15.41	11.10	1.39 (0.91, 2.12)
**CANVAS**			
≤1 year	21.59	14.93	1.45 (0.88, 2.37)
>1 year and ≤2 years	18.87	9.57	1.97 (1.07, 3.62)*
>2 years and ≤4 years	17.92	12.86	1.38 (0.93, 2.05)
>4 years	15.41	11.10	1.39 (0.91, 2.12)
**CANVAS-R**			
≤1 year	11.17	16.16	0.69 (0.44, 1.09)
>1 year and ≤2 years	12.32	10.79	1.14 (0.69, 1.89)
>2 years and ≤4 years	11.38	13.57	0.83 (0.28, 2.48)
>4 years	–	–	–

Abbreviations: CANVAS, CANagliflozin cardioVascular Assessment Study; CANVAS-R, CANagliflozin cardioVascular Assessment Study–Renal; CI, confidence interval; HR, hazard ratio.

*$p < 0.05$.

ESM Table 4. Effects of canagliflozin on fracture according to timing of last use of randomised treatment prior to the event, in the CANVAS Program

Fracture occurred	Number of participants with an event	Participants with an event per 1000 patient-years		HR (95% CI)
		Canagliflozin	Placebo	
**CANVAS Program**				
On randomised treatment (to the end of treatment)	419	16.38	12.55	1.25 (1.02, 1.54)*
On randomised treatment + within 7 days of treatment discontinuation	424	16.52	12.55	1.26 (1.03, 1.55)*
On randomised treatment + within 30 days of treatment discontinuation	430	16.45	12.46	1.26 (1.03, 1.55)*
On randomised treatment + within 90 days of treatment discontinuation	439	16.47	12.57	1.26 (1.03, 1.54)*
On randomised treatment + to the end of the study	496	15.4	11.93	1.26 (1.04, 1.52)*
**CANVAS**				
On randomised treatment (to the end of treatment)	292	18.55	12.07	1.54 (1.17, 2.02)*
On randomised treatment + within 7 days of treatment discontinuation	296	18.73	12.2	1.54 (1.18, 2.02)*
On randomised treatment + within 30 days of treatment discontinuation	299	18.74	12.03	1.56 (1.19, 2.04)*
On randomised treatment + within 90 days of treatment discontinuation	303	18.79	11.81	1.60 (1.22, 2.09)*
On randomised treatment + to the end of the study	350	16.92	10.94	1.55 (1.21, 1.97)*
**CANVAS-R**				
On randomised treatment (to the end of treatment)	127	11.41	13.06	0.87 (0.62, 1.24)
On randomised treatment + within 7 days of treatment discontinuation	128	11.48	12.93	0.89 (0.63, 1.26)
On randomised treatment + within 30 days of treatment discontinuation	131	11.33	12.91	0.88 (0.62, 1.23)
On randomised treatment + within 90 days of treatment discontinuation	136	11.3	13.38	0.84 (0.60, 1.18)
On randomised treatment + to the end of the study	146	11.42	13.23	0.86 (0.62, 1.19)

Abbreviations: CANVAS, CANagliflozin cardioVascular Assessment Study; CANVAS-R, CANagliflozin cardioVascular Assessment Study–Renal; CI, confidence interval; HR, hazard ratio.

*$p < 0.05$.

ESM Table 5. Effects of canagliflozin versus placebo and each dose of canagliflozin versus placebo on biomarkers of bone metabolism and body weight in the CANVAS Program, CANVAS, and CANVAS-R according to duration of follow-up and use of randomised treatment

	CANVAS Program (canagliflozin vs. placebo)	CANVAS (canagliflozin vs. placebo)	CANVAS-R (canagliflozin vs. placebo)	p interaction[a]	CANVAS (100 mg vs. placebo)	(300 mg vs. placebo)	p 100 mg vs. 300 mg[b]
**Mean difference (95% CI) from baseline to 130 weeks of follow-up[c]**							
Serum calcium (mmol/l)	0.008 (0.004, 0.012)*	0.012 (0.006, 0.018)*	0.006 (0.001, 0.011)*	0.14	0.009 (0.001, 0.016)*	0.015 (0.008, 0.022)*	0.08
Serum phosphorous (mmol/l)	0.003 (-0.004, 0.009)	0.010 (0.0002, 0.020)*	-0.003 (-0.011, 0.006)	0.06	0.007 (-0.005, 0.018)	0.014 (0.002, 0.025)*	0.26
Serum magnesium (mmol/l)	0.051 (0.048, 0.055)*	0.069 (0.064, 0.074)*	0.040 (0.036, 0.044)*	<0.001*	0.060 (0.054, 0.065)*	0.078 (0.073, 0.084)*	<0.001*
Alkaline phosphatase (U/l)	-0.283 (-1.621, 1.056)	-1.093 (-3.072, 0.886)	0.220 (-1.585, 2.025)	0.33	-1.828 (-3.581, -0.075)*	-0.355 (-3.011, 2.300)	0.22
Body weight (kg)	-2.460 (-2.662, -2.257)*	-2.674 (-3.007, -2.341)*	-2.321 (-2.575, -2.067)*	0.09	-2.418 (-2.801, -2.036)*	-2.936 (-3.322, -2.551)*	0.007*
**Mean difference (95% CI) from baseline to the last measurement on randomised treatment[c]**							
Serum calcium (mmol/l)	0.012 (0.008, 0.016)*	0.014 (0.007, 0.021)*	0.010 (0.006, 0.015)*	0.33	0.015 (0.007, 0.022)*	0.014 (0.006, 0.022)*	0.91
Serum phosphorous (mmol/l)	0.020 (0.014, 0.026)*	0.017 (0.007, 0.028)*	0.022 (0.014, 0.030)*	0.49	0.010 (-0.002, 0.022)	0.024 (0.012, 0.037)*	0.02*
Serum magnesium (mmol/l)	0.080 (0.077, 0.083)*	0.079 (0.074, 0.084)*	0.081 (0.077, 0.085)*	0.56	0.067 (0.061, 0.073)*	0.091 (0.085, 0.097)*	<0.001*
Alkaline phosphatase (U/l)	-0.303 (-1.452, 0.845)	0.413 (-1.774, 2.599)	-0.796 (-2.008, 0.416)	0.33	-0.544 (-2.121, 1.033)	1.393 (-1.497, 4.284)	0.19
Body weight (kg)	-2.738 (-2.963, -2.513)*	-3.085 (-3.507, -2.662)*	-2.516 (-2.762, -2.271)*	0.02*	-2.779 (-3.263, -2.296)*	-3.399 (-3.887, -2.911)*	0.01*
**Mean difference (95% CI) from baseline to the last measurement regardless of treatment[c]**							
Serum calcium (mmol/l)	0.006 (0.002, 0.010)*	0.015 (0.008, 0.021)*	0.0001 (-0.005, 0.005)	<0.001*	0.015 (0.008, 0.023)*	0.014 (0.006, 0.022)*	0.72
Serum phosphorous (mmol/l)	-0.007 (-0.013, -0.0001)*	0.008 (-0.003, 0.018)	-0.016 (-0.025, -0.008)*	<0.001*	0.006 (-0.006, 0.018)	0.009 (-0.003, 0.022)	0.58
Serum magnesium (mmol/l)	0.033 (0.029, 0.036)*	0.062 (0.056, 0.067)*	0.013 (0.009, 0.017)*	<0.001*	0.054 (0.047, 0.060)*	0.070 (0.063, 0.076)*	<0.001*

Alkaline phosphatase (U/l)	0.264 (-1.181, 1.709)	-0.917 (-3.227, 1.392)	1.010 (-0.840, 2.861)	0.19	-1.450 (-3.671, 0.771)	-0.381 (-3.308, 2.546)	0.46
Body weight (kg)	-2.403 (-2.639, -2.167)*	-2.545 (-2.988, -2.103)*	-2.309 (-2.566, -2.053)*	0.33	-2.301 (-2.809, -1.793)*	-2.796 (-3.307, -2.285)*	0.06

Abbreviations: CANVAS, CANagliflozin cardioVascular Assessment Study; CANVAS-R, CANagliflozin cardioVascular Assessment Study–Renal; CI, confidence interval; HR, hazard ratio.

[a]p interaction between CANVAS and CANVAS-R.

[b]p for trend among placebo, canagliflozin 100 mg, and canagliflozin 300 mg groups in CANVAS.

[c]The mean treatment difference of canagliflozin compared to placebo in the least-squares means and associated 95% CIs were estimated from an analysis of covariance (ANCOVA) model with treatment as an independent effect and adjusting for trial and baseline value.

*p <0.05.

ESM Fig. 1 CANVAS Program: trial flow chart

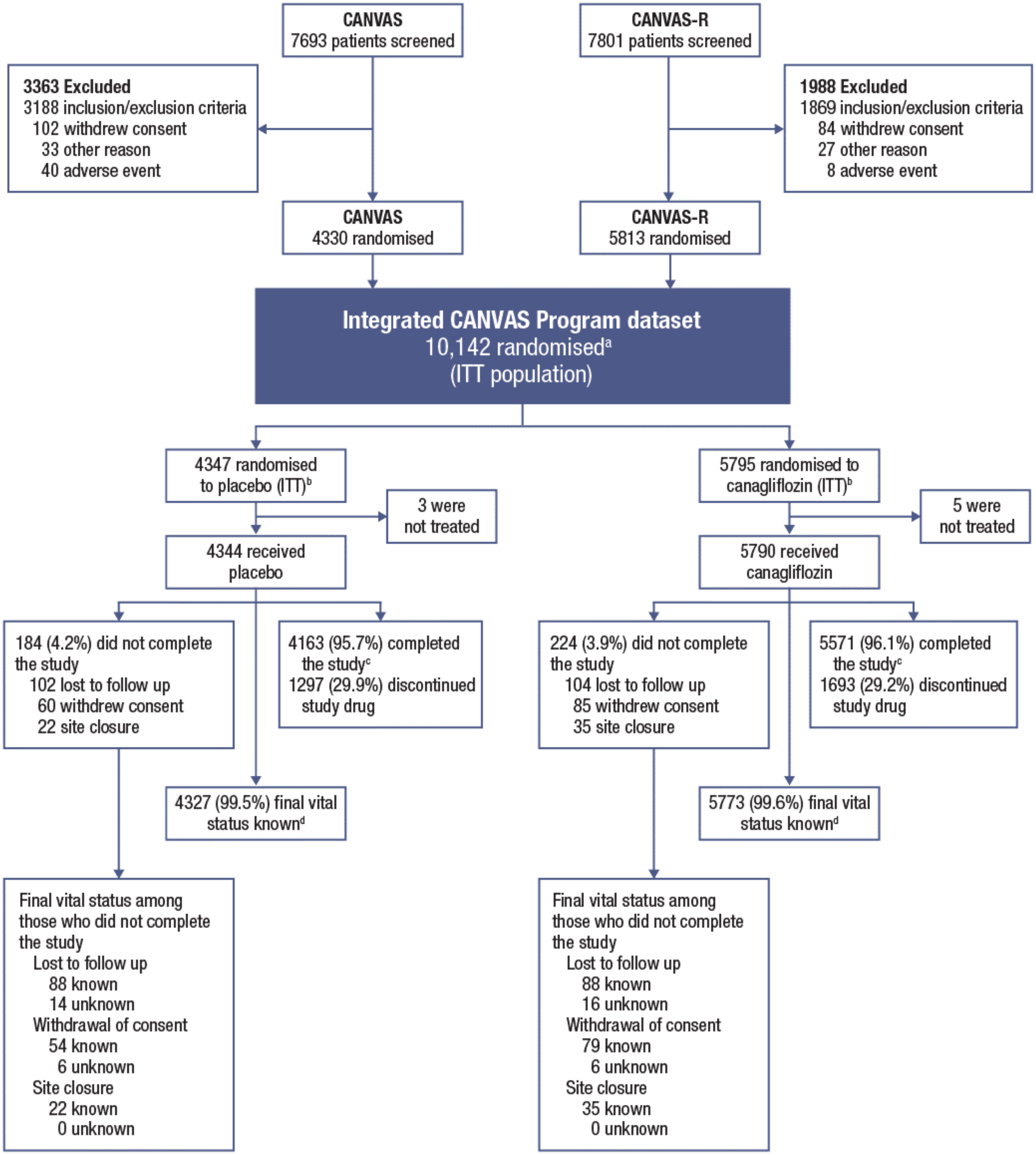

Abbreviations: CANVAS, CANagliflozin cardioVascular Assessment Study; CANVAS-R, CANagliflozin cardioVascular Assessment Study–Renal; ITT, intent-to-treat.

[a]One patient was randomised at 2 different sites and therefore the second randomised ID was excluded from the ITT analysis set.

[b]Percentages calculated based on the ITT analysis set.

[c]A patient is considered as having completed the study, regardless of whether the patient is on or off study drug, if the patient is followed until a time point between the notification of the trial end date (November 1, 2016) and the trial end date (February 23, 2017), or until the time of death for those who died prior to the trial end date.

[d]Including results from the search of public records.

Reprinted from New England Journal of Medicine, Neal B, et al., Canagliflozin and Cardiovascular and Renal Events in Type 2 Diabetes, Volume 377, Pages 644-647 © Massachusetts Medical Society. Reprinted with permission from Massachusetts Medical Society.
